# Use Test of Automated Machine Learning in Cancer Diagnostics

**DOI:** 10.3390/diagnostics13142315

**Published:** 2023-07-08

**Authors:** Manfred Musigmann, Nabila Gala Nacul, Dilek N. Kasap, Walter Heindel, Manoj Mannil

**Affiliations:** University Clinic for Radiology, University Hospital Muenster, WWU Muenster, Albert-Schweitzer-Campus 1, DE-48149 Muenster, Germany

**Keywords:** machine learning, AutoML, radiomics, MRI

## Abstract

Our aim is to investigate the added value of automated machine learning (AutoML) for potential future applications in cancer diagnostics. Using two important diagnostic questions, the non-invasive determination of IDH mutation status and ATRX status, we analyze whether it is possible to use AutoML to develop models that are comparable in performance to conventional machine learning models (ML) developed by experts. For this purpose, we develop AutoML models using different feature preselection methods and compare the results with previously developed conventional ML models. The cohort used for our study comprises T2-weighted MRI images of 124 patients with histologically confirmed gliomas. Using AutoML, we were able to develop sophisticated models in a very short time with only a few lines of computer code. In predicting IDH mutation status, we obtained a mean AUC of 0.7400 and a mean AUPRC of 0.8582. ATRX mutation status was predicted with very similar discriminatory power, with a mean AUC of 0.7810 and a mean AUPRC of 0.8511. In both cases, AutoML was even able to achieve a discriminatory power slightly above that of the respective conventionally developed models in a very short computing time, thus making such methods accessible to non-experts in the near future.

## 1. Introduction

Artificial intelligence (AI) techniques and their applications are an important current topic in medical research. In recent years, medical research in the field of AI has increased enormously [1]. Many corresponding applications have already become an indispensable part of everyday medical life. Machine learning (ML) methods are an important subcategory of artificial intelligence. These techniques attempt to mimic human learning using suitable algorithms. Basically, ML refers to the use and development of computer systems that are able to learn autonomously using suitable algorithms in order to analyze patterns in data and draw appropriate conclusions. In our current study, we focus on so-called “supervised machine learning”, or more precisely on “automated supervised machine learning”. “Supervised” means that these algorithms are first trained with known data and associated outcomes to be predicted in order to recognize relevant facts for the decisions to be made later. Subsequently, supervised machine learning algorithms are able to apply the previously learned logic accordingly to unknown data and thus make decisions independently. “Deep learning”, in turn, is an important subcategory of supervised machine learning. These algorithms use artificial neural networks, which often contain many layers. They are usually trained with a large amount of data.

Non-invasive methods are of great importance for the gentle treatment of patients. This applies, for example, to tumor diagnostics and, in particular, to tumors of the central nervous system (CNS). Artificial intelligence techniques and especially machine learning algorithms offer an enormous potential for this important challenge. Quantitative factors extracted from conventional MRI and CT images (so-called radiomic features) can be used in conjunction with machine learning algorithms to answer important diagnostic questions in the diagnosis of brain tumors in a fully automated, objective, highly precise, and, above all, non-invasive way. For example, Ari et al. showed that radiomic-based machine learning can be used to non-invasively predict pseudoprogression in high-grade gliomas [2]. Krähling et al. developed an MRI-based radiomics model to predict mitotic cycles in intracranial meningiomas before surgery [3]. In Musigmann et al., it was shown that ML algorithms can predict possible total and subtotal resections of skull meningiomas using pre-treatment T1 post-contrast MR images [4].

These and many other studies demonstrate the enormous potential of such techniques. However, as promising as these techniques using ML algorithms may be, it is often difficult for non-experts to apply them correctly. The preparation of data, the selection of features to be included in a model, the determination of a model algorithm suitable for the respective problem, and the optimization of the parameters included in the model often require a considerable amount of expert knowledge. All these and many other steps are difficult to apply correctly and, above all, are often very time-consuming. Moreover, the number of experts capable of developing stable, high-performance models is very limited. This is where the idea of the next “generation” of machine learning comes in, the so-called “automated machine learning” (AutoML). AutoML is a novel tool in the broad field of AI. A major goal of AutoML is to simplify and automate numerous steps in model development to make ML algorithms also accessible to non-experts. But the high level of automation also offers numerous potential advantages for experts. For example, AutoML can be used to get a quick overview of which model algorithms might be promising for further detailed analysis.

Despite the rapidly increasing demand for this comparatively young technology, there have been relatively few publications on automated machine learning in the medical sector. Ikemura et al. used AutoML to predict patients’ chances of surviving a SARS-CoV-2 infection [5]. Karaglani et al. produced predictive biosignatures that provide opportunities for minimally invasive blood-based diagnostic tests for Alzheimer’s disease [6]. Ou et al. used AutoML for intracranial aneurysm treatment outcome prediction and compared the results with those obtained with multivariate logistic regression and a manually developed ML model [7]. Touma et al. developed a completely code-free AutoML model with very high accuracy for classifying cataract surgery phases from videos [8]. Initial studies like these show the great potential that current AutoML algorithms already offer. However, there have been few efforts to apply these techniques in the health sector yet [9,10]. A good overview of the basic methodology, the state of the art, and the possibilities of automated machine learning in healthcare is given by Waring et al. [9].

In this study, we address the question of how powerful and timesaving AutoML algorithms currently are. Is it already feasible to use automated machine learning algorithms to develop reliable models for important questions in medical diagnostics? Are we able to develop models in a much shorter time that nevertheless show comparable performance to models developed in a conventional way? How much expert knowledge is still necessary, which steps can be simplified, and how stable are the models obtained? We analyze these issues using an important task in cancer diagnostics that has already been studied in numerous medical publications using conventional (non-automated) machine learning algorithms as well as deep learning: the automatic classification of brain tumors according to the WHO classification of CNS tumors [11,12,13,14,15,16].

For our analyses performed in this study, we rely on the latest version of the WHO classification for CNS tumors, which was recently published [17,18,19]. In this 2021 version of the WHO classification, tumors are even more consistently subdivided into molecularly and biologically defined entities. Diffuse gliomas of the adult type are primarily divided into the three main tumor types: “astrocytoma, IDH mutated”, “oligodendroglioma, IDH mutated and 1p/19q co-deleted” and “glioblastoma, IDH wild type”. A seven-step diagnostic algorithm can now be used to accurately classify diffuse gliomas of the adult type [20]. The first two of these seven stages are particularly important. In these first two stages, mutations in isocitrate dehydrogenase (IDH) genes IDH1 and IDH2 and mutation/loss of alpha-thalassemia/mental retardation syndrome X-linked (ATRX) expression are considered. A distinction is made between “IDH mutant” and “IDH wild type” (first stage) and between “Nuclear ATRX retained” and “Nuclear ATRX lost” in the second stage. Using this particularly important task, we would like to conduct a realistic use test of AutoML. To enable this, we have previously predicted both IDH and ATRX status with conventional machine learning algorithms. However, it should be explicitly noted that the purpose of our analyses here is not primarily to be able to predict IDH and ATRX status with AutoML particularly accurately. Rather, we want to analyze how easy/difficult or time-consuming it is to achieve comparable results with AutoML as with conventional ML algorithms in order to determine the possible added value of AutoML. Our main aim is to investigate whether non-experts can already use AutoML to develop similarly good models for applications in medical diagnostics as technically skilled developers of conventional machine learning models.

## 2. Materials and Methods

This single-center study was performed in compliance with the Declaration of Helsinki and approved by the local ethics committee (Ärztekammer Westfalen Lippe and University of Münster, 2021-596-f-S). Due to the retrospective nature of the study, written informed consent was waived by the Ärztekammer Westfalen Lippe and University of Münster.

We retrospectively searched our database for patients diagnosed with glioma between June 2008 and April 2021. Initially, 136 patients were screened using the T2 sequence, of whom 12 had to be excluded from our study because the IDH mutation status and/or the ATRX status were unknown. IDH1 and 2 mutation status was obtained according to Yan et al. [21]. The ATRX gene provides instructions for making a protein that plays an essential role in normal development. Its mutation status was analyzed using immunohistochemistry. Our final study cohort of 124 patients was divided into 55 females/69 males, or 43/81 patients with IDH mutation status wild type/mutated, or 75/49 patients with ATRX status retained/lost. The demographic characteristics of the patients in our study cohort are summarized in Table 1 in relation to IDH mutation status and correspondingly in Table 2 in relation to ATRX status.

**Figure 1:**
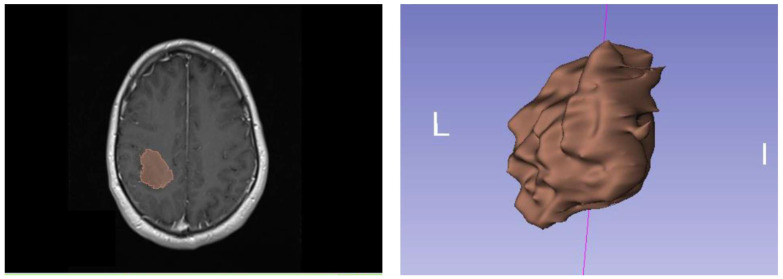
(**Left**): Oligodendroglioma of the right hemisphere, IDH-mutant, ATRX retained, 1p/19q-codeleted, WHO grade 2021: 2 or 3; (**Right**): Semi-automatic segmentation with 3D Slicer.

We performed the segmentation of the contrast-enhancing parts of the tumors semi-automatically using the open-source software platform 3D Slicer (version 4.10, www.slicer.org, accessed on 1 June 2023). Figure 1 shows an example of semi-automatic segmentation with 3D Slicer for an oligodendroglioma (IDH-mutant, ATRX retained, 1p/19q-codeleted). For each of the 124 patients, 107 radiomic features were extracted from the corresponding MRI images by hand-delineated regions of interest (ROI). Radiomic features are reproducibly quantified information derived from image morphological criteria calculated with suitable mathematical algorithms. A good overview of radiomic features and their significance is given by van Griethuysen et al. [22]. By means of radiomic features, for example, the determination of tumor patterns and characteristics can be facilitated. In addition to classical parameters such as tumor diameter or tumor volume, other properties such as shape or heterogeneity within the tumor can also be determined as quantitative features, enabling a non-invasive characterization of tumor-suspicious lesions. To extract the 107 radiomic features from the MRI images, we used the open-source platform PyRadiomics. The PyRadiomics package is available as an implementable plugin for the 3D Slicer platform. The total of 107 radiomic features we used for our analyses is composed of 18 first-order statistics features, 14 shape-based features, 24 gray-level co-occurrence matrix features, 16 gray-level run-length matrix features, 16 gray-level size zone matrix features, 5 neighboring gray-tone difference matrix features, and 14 gray-level dependency matrix features. The exact calculation method for each feature can be found on the PyRadiomics homepage (pyradiomics.readthedocs.io). In addition to the 107 radiomic features, our database contained the gender and age of the patients at the time of diagnosis. Beyond that, no other features are used. The features used therefore consist largely of radiomic features. All features were z-score transformed and subjected to a 95% correlation filter to account for redundancy between the features.

To evaluate the performance achieved with AutoML, we have previously developed conventional ML models for both IDH mutation status prediction and ATRX status prediction. The best machine learning models that we calculated beforehand were obtained using Lasso (least absolute shrinkage and selection operator) regression. We also tested many other conventional machine learning algorithms, such as random forest, bagged trees, naive Bayes, linear discriminant analysis (LDA), support vector machines (SVMs), XGBoost, and a neural net. However, the best results overall were obtained with Lasso regression (for both IDH mutation status prediction and ATRX status). The results obtained with XGBoost were also promising and very close to those obtained with Lasso regression. Currently, there are several software packages such as AutoXGboost and Remix AutoML for automated machine learning. The individual applications usually differ in their degree of automation, their optimization speed, the options for feature preparation and preselection, and the machine learning algorithms contained. Our aim was to test an open-source application that is accessible to everyone. We decided to use “H2O AutoML”, the automated machine learning package, which is available on CRAN. However, it should be explicitly noted here that in addition to the open-source version “H2O AutoML”, a commercial version “H2O Driverless AI” is also available, which contains additional tools. We particularly liked the special feature of the open-source application H2O AutoML which allows the number of models to be trained and the time required for this to be explicitly specified. However, in our view, one disadvantage of H2O AutoML is that the possibilities for feature preparation and suitable preselection of features are still limited, at least for the moment. Therefore, for this use test, we also tested different methods for feature preselection in combination with AutoML. Specifically, we tested AutoML without feature preselection, as well as in combination with Lasso regression and recursive feature elimination (RFE) for feature preselection (see, for example, Darst et al. [23]).

Following the documentation, H2O AutoML simultaneously optimizes models belonging to different algorithm classes: a fixed grid of GLMs (generalized linear models), five pre-specified H2O GBMs (gradient boosting machines) and a random grid of H2O GBMs, three pre-specified XGBoost GBMs and a random grid of XGBoost GBMs, a default random forest (DRF), an extremely randomized forest (XRT), a near-default deep neural net (DNN), and a random grid of deep neural nets [24]. In addition, H2O AutoML uses combinations of these base algorithms, so-called “Stacked Ensemble Models (SEMs)”. In our opinion, these ensemble learners (SEMs) have the advantage that they can further strengthen the model quality, but at the same time, they have the disadvantage that the comprehensibility of the models can suffer in the sense of a black-box character. For our use test, we used all AutoML algorithms except XGBoost GBMs. The functionality of AutoML is already very extensive and is apparently still being further developed. We therefore explicitly refer to the respective current documentations of AutoML, as the numerous possibilities can only be hinted at here [24].

### Statistical Analysis

We performed our statistical analysis using R software (version 4.1.2). The main packages used were “caret” and “xgboost” for the conventional machine learning models computed for comparison purposes and “H2O” for automated machine learning. We randomly divided the final cohort of 124 patients into training data and independent test data using a stratified ratio of 80:20 with a balanced distribution of IDH mutation status and ATRX status, respectively (compare Table 1 and Table 2). All features used for model development were first subjected to a 95% correlation filter to account for redundancies between features. Subsequently, three methods for possible feature preselection in combination with AutoML were tested and compared. In the first method, all remaining features were used for further modeling with AutoML. The second method used Lasso regression in combination with the “VarImp” function. The VarImp function determines the performance gain/loss by adding/removing a specific feature in relation to a given model and determines which features are particularly important in this way. In this second method, we developed models with an increasing number of the most important features and then determined the optimal number of features to include based on the performance achieved (compare Musigmann et al. [25]). Finally, in the third method, we determined the most promising features using RFE. RFE was performed using the random forest algorithm. The algorithm was simultaneously used to determine the optimal number of features to be used. The three methods for possible feature preselection differ in the sense that in the first method, neither the most promising features nor the number of features to be included in the model are determined based on the training data before the modeling process is initiated with the conventional ML algorithms or with AutoML. In the second method (using Lasso regression), the most promising features are first identified, but the exact number of features to be included is then determined in a separate second step. In this second step, the number of features included is further increased until an optimal number is reached in terms of model performance. In the third method (RFE), on the other hand, the most promising features and their optimal number are determined in a single step.

**Figure 2:**
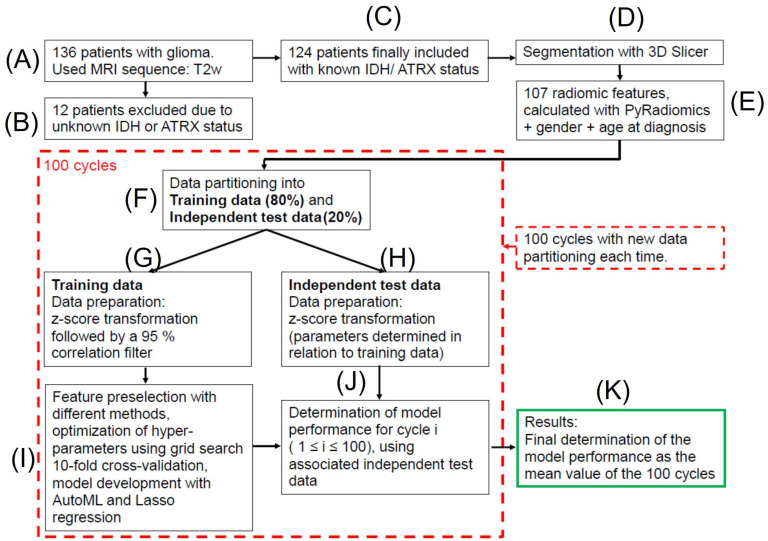
Flowchart describing the methodological approach. Each model (AutoML with different feature preselection methods and Lasso regression models) is developed 100 times, each time with a new data partitioning. For each of these 100 individual cycles, the preselection of the features, the model development (training), and the determination of the hyperparameters included in the models were performed using the associated individual training data. The hyperparameters were determined using a grid search 10-fold cross-validation in the case of conventional machine learning models and completely automatically in the case of using AutoML (again for each of the 100 individual cycles). The performance of each model is first determined for each individual cycle using the associated independent test data (resulting in a total of 100 individual performance values). Subsequently, the final model performance is calculated as the average of the 100 cycles. Simply put, we fully developed each model with 100 different data partitions 100 times and then calculated the average performance values from those 100 cycles. Steps (**A**–**E**,**K**) are performed only once. Steps (**F**–**J**), on the other hand, are performed 100 times.

The preselection of the features, the model development (training), and the determination of the hyperparameters included in the models were performed using the training data. The hyperparameters were determined using a grid search with 10-fold cross-validation in the case of conventional machine learning models and completely automatically in the case of using AutoML. The discriminatory power of the models was subsequently determined using the unknown/independent test data. Each model was 100 times completely developed and subsequently tested, with a new split of training data and independent test data in each of these cycles. This step was conducted to exclude random effects related to the data partitioning used. All performance values were calculated as the average of these 100 cycles, with the individual results for each of these cycles determined using the associated independent test data. This procedure is described in detail in Musigmann et al. [25]. For a better understanding, we have also summarized the entire process in a flowchart in Figure 2.

The performance achieved with the different modeling approaches was compared using the area under the curve (AUC) of the receiver operating characteristic (ROC). It should be noted that in this study we do not use other commonly used metrics such as accuracy, sensitivity, and specificity. We do not use these metrics in this study because they are highly dependent on the threshold used to distinguish the classes to be predicted. This is especially true in cases with a strong imbalance between the two classes to be predicted, which is the case for both distributions analyzed in our study (compare Table 1 and Table 2). One approach to dealing with imbalanced data is to use so-called oversampling and undersampling techniques such as SMOTE (synthetic minority over-sampling technique) [26,27] and ROSE (random over-sampling examples) [28,29]. These techniques are of particular interest in classification problems where one class is significantly less frequently occupied compared to the other class(es). A simple way to correct such an imbalance is to remove records assigned to the more populated class(es) from the data or, alternatively, duplicate records assigned to the less populated class. Similar options are also available for H2O AutoML. Here, the “balance_classes” option can be used to balance class distributions. When enabled, H2O either undersamples the majority classes or oversamples the minority classes [24]. We tested these techniques extensively in our study, but they did not significantly change our results. Therefore, to ensure that the methodological approach used for our conventional machine learning models and for AutoML is as comparable and simple as possible, we decided not to use such oversampling and undersampling techniques in our study. In our study, we consider the area under the precision-recall curve (AUPRC) instead of accuracy, sensitivity, or specificity. This is a metric that is particularly suitable for unbalanced data. However, it should be noted that AutoML offers many possibilities to optimize the confusion matrix (which summarizes the classification results) in terms of accuracy, sensitivity, specificity, and numerous other metrics such as precision, recall, the F1 score, and Matthew’s correlation coefficient (MCC).

When running AutoML, numerous different algorithms are trained simultaneously. Therefore, the best of these models must subsequently be determined using a suitable metric (the so-called “sort metric”). We tested different metrics (AUC, AUPRC, log loss, mean per class error) and then decided to use AUPRC to account for the imbalance in our data. It should be noted, however, that the influence of the sorting metric used on the achieved discriminatory power of our final models was rather small, at least with respect to the sorting metrics we tested.

For the sake of clarity, we have summarized the most important methodological points and algorithms used in our study once again in Table 3. The table also lists all the conventional machine-learning algorithms we tested. As described, all steps listed in Table 3 are performed 100 times for each model tested. A new data partition is used in each cycle. The independent test data is used only to determine model performance. The final model performance is determined as the average of these 100 runs.

## 3. Results

### 3.1. Finding the Best Class of Model Algorithms with AutoML

At the beginning of our analyses with AutoML, we initially used all classes of model algorithms (i.e., “GLM”, “DRF”, “GBM”, “DeepLearning”, and “StackedEnsemble”) for model training with the exception of the XGBoost GBM models. For both IDH mutation status prediction and ATRX status prediction, we initially performed 100 runs each with different splits of the data into training and test data. In the case of IDH mutation status prediction, AutoML mainly chose GBM algorithms. For the prediction of the ATRX status, on the other hand, mainly deep-learning algorithms were selected. This selection was also only subordinately dependent on the sorting metric used to determine the best model algorithm in each case. However, during these initial trials, we quickly realized that the algorithm classes selected by AutoML based on the training data (i.e., “GBM” and “DeepLearning”) did not necessarily also provide the best results in terms of the associated independent test data. The deep-learning algorithms and the stacked ensembles even performed the worst of all algorithms on both optimization problems in terms of the performance achieved with the independent test data. The fact that the deep-learning algorithms performed rather poorly in our experiments is certainly partly due to the comparatively small number of data sets included in our study cohort. However, this indicates that AutoML does not necessarily find the most suitable algorithm class for a particular optimization problem on its own.

Therefore, in a second experiment, we fixed each of the possible algorithm classes once, then performed again 100 runs each, and finally compared the results with respect to the independent test data. This approach has the advantage that it can also be used to test algorithms that AutoML would otherwise not or only rarely select. Conversely, this approach is also suitable for quickly excluding classes of algorithms (such as “DeepLearning” in our case) that may even require long optimization times but may prove to be suboptimal when subsequently tested with independent data. In this way, the computing time invested can be used to optimize the truly promising algorithms.

Applying the described approach to determine the most promising class of algorithms, we found in the case of IDH mutation status prediction that the highest performance using the independent test data was indeed achieved with GBM algorithms. As described, in the case where no specifications were made, AutoML also selected this algorithm class most frequently on its own. Without specifications, AutoML has also selected the algorithm classes “DRF”, “DeepLearning”, and “StackedEnsemble” in rare cases. However, our tests also showed that models developed with GLM algorithms performed only slightly worse than models developed with GBM algorithms. This is interesting in the sense that AutoML practically never automatically selected this class of algorithms.

Next, we determined the most promising class of algorithms for predicting ATRX status. As already described, AutoML without specifications selected deep-learning algorithms almost without exception, but these performed particularly poorly on the dependent test data. However, when testing all classes of algorithms, we quickly found that the GLM algorithms were the most promising for predicting ATRX status. This is remarkable because, in our very elaborate search for the most suitable conventional ML algorithm, we finally found a Lasso regression. Lasso regression (together with other algorithms such as ridge regression or an elastic net) belongs to the class of GLM algorithms. Using AutoML, we identified the class of the most suitable algorithms in the shortest possible time and thus saved a lot of time that we had previously spent searching for a suitable conventional ML algorithm.

### 3.2. Prediction of IDH Mutation Status Using AutoML

After having found the most promising class of algorithms for both IDH mutation status prediction and ATRX status prediction, we tested AutoML in combination with the three feature preselection options already described: (1) no feature preselection, i.e., using all features; (2) feature preselection using Lasso regression to determine the most important features; and (3) RFE. In our further analyses, we first considered the IDH mutation status again. We developed corresponding models with AutoML using all three methods for possible feature preselection and then compared the results with the results we were able to achieve with our best conventional machine learning algorithm.

**Figure 3:**
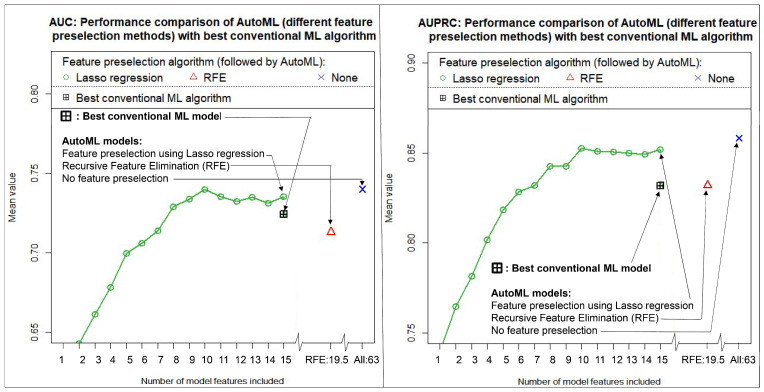
Achieved discriminatory power in predicting IDH mutation status using different feature preselection methods in combination with AutoML as well as a conventional machine learning algorithm. All values were calculated with independent test data and as mean values of 100 cycles. (**Left**): Area under the curve of the receiver operating characteristic (AUC). (**Right**): Area under the precision-recall curve (AUPRC).

The results of our calculations for the prediction of IDH mutation status are summarized in Figure 3. All performance values were calculated with independent test data and as mean values of 100 runs each. The left figure shows the results in terms of AUC achieved, and the right figure in terms of AUPRC. The exact numerical values are listed in Table 4, together with their 95% confidence intervals. The values given in the table for the feature preselection by means of Lasso regression refer to the model with 10 features included. This number of features resulted in the highest discriminatory power. The RFE algorithm, on the other hand, selected slightly more features, with an average of 19.5 features. The third model without a feature preselection algorithm contained 63 features. Finally, the best conventional ML model resulted in 15 features included.

It is very interesting to observe that all three AutoML approaches produced extremely similar results compared to our best conventional ML algorithm, which we had elaborately developed beforehand. However, using AutoML, we needed significantly less time, and nevertheless, we achieved the same discriminatory power. Overall, the AutoML approach without a preceding feature preselection algorithm performed even better. Using independent test data, this approach yielded a mean AUC of 0.7400 [0.5041, 0.9326] and a mean AUPRC of 0.8582 [0.6737, 0.9695] on average (100 cycles), demonstrating high performance in discriminating IDH wild-type from IDH mutated gliomas. The numbers in brackets indicate the 95% confidence interval. Since no feature preselection was performed in this approach, this methodology is particularly simple and, at the same time, extremely fast in terms of the computing time required. We have limited the time to train/optimize each of the AutoML GBM models to a few minutes using the “max_runtime_secs” parameter. In our case, however, we even found that 60 s was sufficient to achieve comparable results and that longer training times did not contribute significantly to a further improvement in discriminatory power.

It should be noted here that we also calculated *p*-values for our final models. For this purpose, we compared our models to a null model using the ANOVA function in R (ANOVA = analysis of variance). By construction, the *p*-values obtained depended slightly on the individual data partitioning during each of the 100 cycles. However, the *p*-values were always smaller than 10 × 10^−6^. We also compared the four final models listed in Table 4 using the DeLong test. No single comparison of the two models showed a significant difference (significance level *p* < 0.05) in terms of the AUCs achieved. However, when comparing the four models with a null model using the DeLong test, again only extremely small *p*-values (*p* < 10 × 10^−6^) were obtained for all four models. Therefore, the likelihood that our final models do not significantly differentiate the two groups of IDH mutation status is extremely low.

In addition to the results already shown and discussed, we calculated the AUC, AUPRC, and several other performance values for our best AutoML model (AutoML without feature preselection) based on all 124 datasets used. We used 5-fold cross-validation. With respect to the validation data, we obtained the following performance results: AUC = 0.7832 [0.0743], AUPRC = 0.8870 [0.0459], accuracy = 0.7740 [0.0733], balanced accuracy = 0.6965 [0.1159], MCC = 0.5617 [0.0947], and F1 score = 0.8352 [0.0605]. The values in brackets indicate the standard deviation of the 5 validation groups in the 5-fold cross-validation. The comparison of performance in terms of AUC and AUPRC shows (see Table 4) that very similar results were obtained with the independent test data as with the validation data. As expected, a slightly higher discriminatory power is achieved with the validation data compared to the independent test data.

### 3.3. Prediction of ATRX Status Using AutoML

Consistent with the prediction of IDH mutation status, we also examined the performance achieved with AutoML in predicting ATRX status. As already described, this time GLM algorithms very quickly proved to be the most promising algorithm class for model development with AutoML. Among the conventional ML algorithms tested, Lasso regression also turned out to be the most suitable algorithm for predicting ATRX status.

After deciding on GLM algorithms for training the AutoML models, we again ran the three different feature preselection procedures. AutoML took only a few seconds to train/optimize a GLM model (this time without setting a maximum calculation time), so despite our 100 repetitions, we were able to calculate the entire model set of 1700 individual models ((15 × Lasso feature preselection resulting in 1 to 15 features + 1 × RFE + 1 × no feature preselection) × 100 = 1700) in only a few hours. In fact, most of the computing time was even spent on feature preselection using Lasso regression or the RFE algorithm, which was performed before the subsequent model optimization with AutoML.

**Figure 4:**
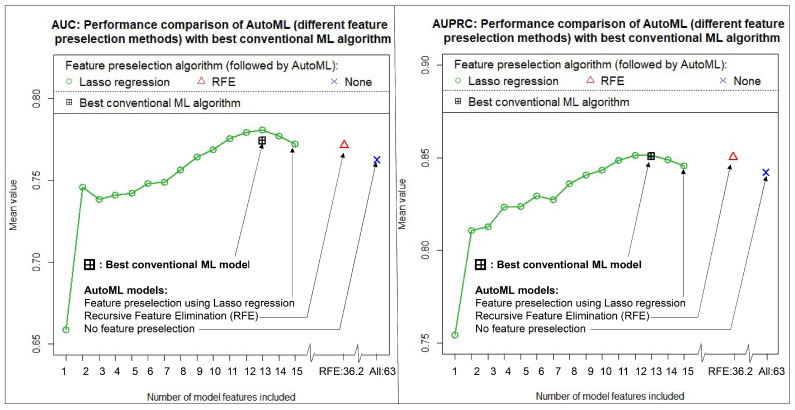
Achieved discriminatory power in predicting ATRX status using different feature preselection methods in combination with AutoML as well as a conventional machine learning algorithm. All values were calculated with independent test data and as mean values of 100 cycles. (**Left**): Area under the curve of the receiver operating characteristic (AUC). (**Right**): Area under the precision-recall curve (AUPRC).

Figure 4 shows the results obtained (again, averaged over 100 runs). In addition, Table 5 summarizes the exact numerical performance values together with their confidence intervals. Once again, all four model approaches tested led to extremely similar performance values. This time, the best results overall were achieved with AutoML in combination with Lasso regression for feature preselection. The highest discriminatory power was achieved here with 13 features included, resulting in a mean AUC of 0.7810 [0.5563, 0.9111] and a mean AUPRC of 0.8511 [0.6603, 0.9522]. The results demonstrate that ATRX status can also be predicted with very high discriminatory power. The number of features in this model corresponds exactly to the number of features determined for the best conventional ML model. This is probably due to the fact that in the case of ATRX status prediction, both approaches (AutoML + conventional ML) used very similar algorithms (i.e., GLMs). In the case where RFE was used for feature preselection, 36.2 features were selected on average. In total (without feature preselection), 63 features were again available. However, in line with the prediction of the IDH mutation status, the method of feature preselection used again had only a minor influence on the discriminatory power achieved.

We also calculated *p*-values for our models predicting ATRX status. Consistent with the prediction of IDH mutation status, the *p*-values of the final models predicting ATRX status were less than 10 × 10^−6^. The pairwise comparison of the four models listed in Table 5 using the DeLong test again showed no significant differences with regard to the AUCs achieved. The biggest difference was found when comparing the two AutoML models where no feature preselection or feature preselection using Lasso regression was performed. However, when comparing the four models with a null model, all four *p*-values again indicated high significance (*p*-values < 10 × 10^−6^). Finally, for our best AutoML model for predicting ATRX status (feature preselection with Lasso regression and 13 features included), we also calculated the AUC, AUPRC, and several other performance values based on all 124 datasets used. Consistent with the approach used to predict IDH mutation status, we again used 5-fold cross-validation. For the validation data, we obtained the following performance results: AUC = 0.8268 [0.0680], AUPRC = 0.8703 [0.0559], accuracy = 0.8230 [0.0659], balanced accuracy = 0.8099 [0.0760], MCC = 0.6316 [0.1400], and F1 score = 0.8594 [0.0505]. The values in brackets again indicate the standard deviation of the 5 validation groups in the 5-fold cross-validation. The comparison of performance in terms of AUC and AUPRC shows (see Table 5) that again very similar results were obtained with the independent test data compared to the validation data.

## 4. Discussion

In this study, we investigated the added value of automated machine learning for potential applications in medical cancer diagnostics. Based on two important diagnostic questions, the prediction of IDH mutation status and ATRX status, we found that it is already possible to develop models using AutoML that have comparable discriminatory power to conventional ML models developed by technically experienced developers. Using AutoML, we were able to significantly reduce the time required to develop these two models compared to conventional approaches and still achieve the same discriminatory power. Furthermore, we believe that the significant simplifications associated with AutoML in the development of ML models already enable non-experts to develop powerful models. The AutoML models we developed required only a few lines of computer code. Numerous algorithms were tested simultaneously in a very short period of time. This is where we currently see the greatest added value of AutoML. We were able to quickly identify promising classes of model algorithms for further model development. This step is often very time-consuming in conventional machine learning. Using AutoML, we were able to concentrate directly on fine-tuning the models. Until now, testing possible conventional ML algorithms required a considerable amount of expert knowledge, e.g., for the appropriate determination of the hyperparameters included in the various model algorithms. However, algorithms such as AutoML represent an essential step towards better meeting the significant demand for ML algorithms for future medical applications. Some other studies have already found comparable evaluations to ours. For example, Antaki et al. assessed the discriminative performance of AutoML in differentiating retinal vein occlusion (RVO), retinitis pigmentosa (RP), and retinal detachment (RD) from normal fundi using ultra-widefield pseudocolor fundus images. They used a publicly available image data set of 2137 labeled images, reviewed the data set for low-quality and mislabeled images, and then uploaded them to the Google Cloud AutoML Vision platform for training and testing. The performance achieved was comparable to deep-learning models derived by AI experts for RVO and RP but not for RD [30]. Abbas et al. evaluated the performance of an AutoML model that predicts visual acuity outcomes in patients receiving treatment for neovascular age-related macular degeneration, in comparison to a manually coded model built using the same dataset. Their AutoML model achieved a highly similar performance to their XGBoost model (AUC-AutoML: 0.849, AUC-XGBoost: 0.847) [31]. Romero et al. compared the performance of different AutoML tools for predicting outcomes for different diseases of interest on datasets with high class imbalance. The AutoML tools showed improvement from the baseline random forest model but did not differ significantly from each other [32]. A further comparison of an AutoML algorithm with conventional algorithms was also carried out by Ou et al. using the example of the predictability of intracranial aneurysm treatment outcomes [7]. They compared the AutoML algorithm with multivariate regression and a random forest model. In this study, the AutoML model even performed significantly better than the two conventional models.

In our study, in addition to testing AutoML, we also analyzed different options for feature preselecting. Interestingly, for both diagnostic questions examined in the study, we found that the feature preselection methodology used had no significant influence on the discriminatory power achieved. We even achieved similar discriminatory power with and without feature preselection. However, there are some important points to note here. First, it is important to mention that, as described, we subjected all features to a 95% correlation filter before training the models to account for redundancies between features. Without the use of the correlation filter, the models proved to be unstable and showed lower discriminatory power. Secondly, it should also be noted that AutoML does not actually use all the features offered for training in the final models. In the case of GLM models, for example, the number of effectively used features is limited by regularization techniques. Thirdly, we trained our models with only 100 data sets each and a maximum of 63 possible features. However, if, for example, databases with considerably more data sets and features are available, algorithms for feature preselection are of great importance, as otherwise, very long computing times may become necessary very quickly. Overall, the fact that feature preselection, with the exception of the correlation filter used, was not beneficial in our two optimization problems studied should therefore not be generalized.

Regardless of the added value of H2O AutoML specifically investigated in this study, it is generally important in automated machine learning to find an appropriate combination of feature preprocessing, feature preselection, model selection, and hyperparameter tuning. Due to the many factors to be taken into account, this task can quickly become very complex and computationally intensive. The combinations of several of these individual steps are also called “pipelines”. Accordingly, so-called “pipeline optimizers” are systems that support the automation of several machine learning steps. Two of these optimization algorithms are TPOT (tree-based pipeline optimization Tool) [33] and Auto-Sklearn [34]. As an example of the application of such techniques, Dafflon et al. used TPOT to conduct a genetic programming-based approach to find a model and its hyperparameters that more closely predicts the brain age from cortical anatomical measures [35].

Our study has some limitations. First, the retrospective character and the fact that it is a single-center study should be mentioned. In addition, we had to exclude 12 patients due to unknown IDH mutation status and/or ATRX status. Finally, the data set we used included only 124 patients. To obtain the most reliable results, we have therefore fully developed each of our models 100 times and then determined the performance and its variability (confidence intervals) with the corresponding independent test data. However, to be able to analyze the performance of AutoML even more precisely, it would be advantageous to relate corresponding analyses to even larger study cohorts. Regardless of these limitations, this study shows that AutoML algorithms can significantly simplify and automate numerous steps in the model development of conventional ML algorithms, making such methods also accessible to non-experts. But AutoML also offers considerable advantages for experts, as these techniques can be used to develop high-performance models in much less time than before.

## 5. Conclusions

The demand for artificial intelligence and machine learning techniques for potential medical applications is increasing rapidly. In order to meet this demand as effectively as possible, it is important to simplify these techniques to make them also accessible to non-experts. Such a possible and very promising considerable simplification can be achieved by means of automated machine learning algorithms. We have investigated such an algorithm using two clinical case studies: the prediction of IDH mutation status and ATRX status, which are two very important markers in cancer diagnostics. With only a few lines of computer code, we were able to develop models in a minimum amount of time that nevertheless have comparable discriminatory power to conventional machine learning models but whose development required a considerable amount of expert knowledge.

## Figures and Tables

**Table 1:** Demographic characteristics of patients used to test AutoML in terms of predictability of IDH mutation status. The MR images were acquired using the T2 sequence.

	Training Data	Independent Test Data	Total Data
Number of patients	100	24	124
Gender (in %)			
Female	44.55	43.54	44.35
Male	55.45	56.46	55.65
Mean age (in years)	43.67	42.87	43.52
IDH mutation status (in %)			
Wild type (not mutated)	35.00	33.33	34.68
Mutated	65.00	66.67	65.32

**Table 2:** Demographic characteristics of patients used to test AutoML in terms of predictability of ATRX status. The MR images were acquired using the T2 sequence.

	Training Data	Independent Test Data	Total Data
Number of patients	100	24	124
Gender (in %)			
Female	44.03	45.71	44.35
Male	55.97	54.29	55.65
Mean age (in years)	43.54	43.44	43.52
ATRX status (in %)			
Retained (not mutated)	60.00	62.50	60.48
Lost (mutated)	40.00	37.50	39.52

**Table 3:** Summary of the most important methodological points and algorithms used.

Data partitioning:
80% training data/20% independent test data
Feature preparation:
z-score transformation
95% correlation filter
ML algorithms used (trained with training data):
Lasso regression (best conventional algorithm)
Naive Bayes
LDA
Random forest
Bagged trees
SVM (linear)
SVM (radial)
SVM (polynomial)
Neural net
XGBoost
AutoML (see text for included algorithms)
Tuning of hyperparameters (based on training data):
10-fold cross-validation
Performance metrics used:
AUC, AUPRC
Sort metric used (only relevant in case of AutoML):
AUPRC
Determination of model performance:
Based on independent test data

**Table 4:** Achieved discriminatory power in predicting IDH mutation status using different feature preselection methods in combination with AutoML as well as a conventional machine learning algorithm. All values were calculated with independent test data and as mean values of 100 cycles. The numbers in brackets indicate the 95% confidence interval. All hyperparameters included in the models were optimized using 10-fold cross-validation (see description in the text). AUC: Area under the curve of the receiver operating characteristic. AUPRC: area under the precision-recall curve. ^1^ On average, the RFE algorithm selected 19.5 features during the 100 cycles.

Algorithm	AUC	AUPRC
Feature preselection with Lasso regression (10 features) + AutoML	0.7400 [0.5510, 0.9059]	0.8524 [0.7211, 0.9527]
Recursive Feature Elimination (19.5 features ^1^) + AutoML	0.7128 [0.4983, 0.8984]	0.8319 [0.6571, 0.9540]
No feature preselection (63 features) + AutoML	0.7400 [0.5041, 0.9326]	0.8582 [0.6737, 0.9695]
Conventional ML model (15 features)	0.7242 [0.4889, 0.9248]	0.8317 [0.6132, 0.9646]

**Table 5:** Achieved discriminatory power in predicting ATRX status using different feature preselection methods in combination with AutoML as well as a conventional machine learning algorithm. All values were calculated with independent test data and as mean values of 100 cycles. The numbers in brackets indicate the 95% confidence interval. All hyperparameters included in the models were optimized using 10-fold cross-validation (see description in the text). AUC: Area under the curve of the receiver operating characteristic. AUPRC: Area under the precision-recall curve. ^1^ On average, the RFE algorithm selected 36.2 features during the 100 cycles.

	Performance Metric	
Algorithm	AUC	AUPRC
Feature preselection with Lasso regression (13 features) + AutoML	0.7810 [0.5563, 0.9111]	0.8511 [0.6603, 0.9522]
Recursive Feature Elimination (36.2 features ^1^) + AutoML	0.7715 [0.5965, 0.9252]	0.8502 [0.6867, 0.9576]
No feature preselection (63 features) + AutoML	0.7628 [0.5630, 0.8998]	0.8422 [0.6778, 0.9428]
Conventional ML model (13 features)	0.7744 [0.5446, 0.9185]	0.8495 [0.6794, 0.9535]

## Data Availability

Available upon reasonable request.
